# A meta-analysis of the performance of the Pima^TM^ CD4 for point of care testing

**DOI:** 10.1186/s12916-015-0396-2

**Published:** 2015-07-25

**Authors:** Lesley E. Scott, Jennifer Campbell, Larry Westerman, Luc Kestens, Lara Vojnov, Luciana Kohastsu, John Nkengasong, Trevor Peter, Wendy Stevens

**Affiliations:** Department of Molecular Medicine and Haematology, Faculty of Health Science, School of Pathology, University of the Witwatersrand, 7 York Road Parktown, Johannesburg, South Africa; Clinton Health Access Initiative, Boston, MA USA; US Centers for Disease Control, Atlanta, GA USA; Department of Biomedical Sciences, University of Antwerp, Antwerp, Belgium; Laboratory of Immunology, Department of Biomedical Sciences, Institute of Tropical Medicine, Antwerp, Belgium; National Health Laboratory Service, Johannesburg, South Africa

**Keywords:** Pima CD4, Point of care testing, Meta-analysis, CD4 misclassification

## Abstract

**Background:**

The Alere point-of-care (POC) Pima™ CD4 analyzer allows for decentralized testing and expansion to testing antiretroviral therapy (ART) eligibility. A consortium conducted a pooled multi-data technical performance analysis of the Pima CD4.

**Methods:**

Primary data (11,803 paired observations) comprised 22 independent studies between 2009–2012 from the Caribbean, Asia, Sub-Saharan Africa, USA and Europe, using 6 laboratory-based reference technologies. Data were analyzed as categorical (including binary) and numerical (absolute) observations using a bivariate and/or univariate random effects model when appropriate.

**Results:**

At a median reference CD4 of 383 cells/μl the mean Pima CD4 bias is -23 cells/μl (average bias across all CD4 ranges is 10 % for venous and 15 % for capillary testing). Sensitivity of the Pima CD4 is 93 % (95 % confidence interval [CI] 91.4 % - 94.9 %) at 350 cells/μl and 96 % (CI 95.2 % - 96.9 %) at 500 cells/μl, with no significant difference between venous and capillary testing. Sensitivity reduced to 86 % (CI 82 % - 89 %) at 100 cells/μl (for Cryptococcal antigen (CrAg) screening), with a significant difference between venous (88 %, CI: 85 % - 91 %) and capillary (79 %, CI: 73 % - 84 %) testing. Total CD4 misclassification is 2.3 % cases at 100 cells/μl, 11.0 % at 350 cells/μl and 9.5 % at 500 cells/μl, due to higher false positive rates which resulted in more patients identified for treatment. This increased by 1.2 %, 2.8 % and 1.8 %, respectively, for capillary testing. There was no difference in Pima CD4 misclassification between the meta-analysis data and a population subset of HIV+ ART naïve individuals, nor in misclassification among operator cadres. The Pima CD4 was most similar to Beckman Coulter PanLeucogated CD4, Becton Dickinson FACSCalibur and FACSCount, and less similar to Partec CyFlow reference technologies.

**Conclusions:**

The Pima CD4 may be recommended using venous-derived specimens for screening (100 cells/μl) for reflex CrAg screening and for HIV ART eligibility at 350 cells/μl and 500 cells/μl thresholds using both capillary and venous derived specimens. These meta-analysis findings add to the knowledge of acceptance criteria of the Pima CD4 and future POC tests, but implementation and impact will require full costing analysis.

## Background

Globally, 34 million individuals are infected with HIV, and currently nearly 14 million worldwide are receiving antiretroviral therapy (ART) [1]. The number of additional HIV-positive patients eligible for ART has increased a further 12 million for a total of 25.9 million eligible patients. The treatment gap, however, remains large and better methodologies or healthcare system changes are required to improve the number of individuals initiating treatment [2]. Many HIV-positive patients, however, do not have reliable access to required diagnostic laboratory tests, including CD4 enumeration since CD4 testing is often only available in regional laboratories. This longer turnaround time on results impacts on patient retention in care [3–5]. It should also be noted that the need for such testing and the thresholds of CD4 counts that clinicians deem relevant for treatment initiation are moving targets [6]. In addition to ART initiation, CD4 counts are also being used as a screening tool for reflex testing to screen for and prevent *Cryptococcal* meningitis in patients with a CD4 count <100 cells/μl [2]. There is therefore a critical need to expand access to HIV diagnostic testing services.

Generally, method comparison studies of new technologies compared to the reference technologies are performed to address these critical issues. The Pima CD4 (Alere, Jena, Germany) was one of the first commercially available point-of-care (POC) CD4 technologies. It entered the market in 2009 and provides a CD4 result in 20 min, is very easy to use, requires no refrigeration of reagents or controls, and can be operated with battery power [7]. Over many years the ART initiation target in many low- and middle-income countries has been CD4 counts <200 cells/μl, expanded more recently to include thresholds of <350 cells/μl [8] and was further raised to <500 cells/μl in the WHO 2013 guidelines [2]. The selection of accurate and affordable POC CD4 technologies that can increase access to testing remains necessary in many regions for attaining ambitious 2015 treatment initiation goals [9]. Implementation of POC CD4 testing in primary health care facilities has been shown to reduce test turnaround time, reduce pre-ART loss to follow-up, and increase prompt ART initiation [10, 11], yet implementing an inaccurate and imprecise CD4 testing platform would be costly to patients and national programs.

Despite the more than 50 technical evaluation studies of the Pima CD4 being performed in dozens of countries, this has not been reported in a consolidated format nor has the venous versus capillary blood detection debate reached a conclusion. Each study adds to the breadth of knowledge, but there is little guidance on acceptable evaluation criteria specifically for CD4 testing technologies [12]. We sought to conduct a pooled data meta-analysis to address these issues and generate guidance for national programs and future CD4 test developers. The objectives of this pooled multi-data analysis were to summarize the performance of the Alere Pima POC CD4 technology at three clinical thresholds [100 cells/μl (to identify patients in need of reflex testing for prevention of *Cryptococcal* meningitis); 350 cells/μl (to identify patients eligible for ART according to the 2010 WHO guidelines) and 500 cells/μl (to identify patients eligible for ART according to the 2013 WHO guidelines)] compared with several laboratory-based reference technologies and across global regions.

## Methods

### Study selection and data pooling

An initiative between researchers at the University of the Witwatersrand, the Clinton Health Access Initiative (CHAI), the World Health Organization (WHO) and the US Centers for Disease Control and Prevention (CDC) led to the formation of a Pima CD4 consortium comprising 34 individuals. Studies were either undergoing publication, already published evaluations on the Pima CD4, or were in-country regulatory evaluations of the technology and were willing to supply their study data. A PRISMA (*Preferred Reporting Items for Systematic Reviews and Meta-Analyses*) analysis was performed with a modified checklist since this “meta-analysis” involved re-analysis of observation pairs from groups willing to supply their data [13]. The STARSD (Standards for Reporting Studies of Diagnostic Accuracy) analysis criteria were followed where applicable to method comparison of CD4 paired observations [14]. Data sets from each group were received in MS Excel format and merged into one worksheet containing the following minimum set of variables: observation pair number, country, Pima CD4 count (cells/μl), reference CD4 count (cells/μl) and type, specimen type (capillary or venous derived) and year in which observations were collected. The “predicate”, “in-country”, “gold standard”, “standard” and “reference” CD4 technology terminology often applied to CD4 enumeration evaluation studies are collectively referred to in this article as reference CD4 technology. These included the Beckman Coulter PanLeucogated CD4 (Beckman Coulter, Miami, FL, USA), the FACSCount, FACSCalibur and FACScan (Becton Dickinson Biosciences, San Jose, CA, USA), the CyFlow (Partec, Munster, Germany) and the Guava EasyCD4 (Merck Millipore, Billerica, MA).

### Statistical analysis

Analyses were performed using MS Excel, Stata 13 (StataCorp, College Station, TX) and SAS (SAS Version 9.2,SAS Institute Inc). Data were analyzed as categorical (including binary) and numerical (absolute) observations and various subset analyses were performed as described in Table 1.

**Table 1:** Description of data analysis

**(a)** Catagorical data analysis	
Data format	Methods
The number (proportion) of CD4 observations in the following CD4 categories: <100 cells/μl; 100 – 350 cells/μl; 350–500 cells/μl and >500 cells/μl was determined for both Pima CD4 and reference methods. The data were further divided into the type of specimen (venous or capillary) tested on the Pima CD4	Significance (p ≤0.05) between categories was determined using the proportions test.
The Pima CD4 and reference CD4 observations were also converted to binary (0 = above the specified threshold and 1 = below the threshold). The observation pairs were also sorted by specimen type, comparator reference technology and year when observations were collected.	The false positive, false negative, sensitivity (ability to correctly identify patients requiring treatment) and specificity (ability to correctly identify patients not requiring treatment) were calculated for the three clinical thresholds of the entire dataset. The total misclassification rate (percentage) was calculated as the addition of false positive rate and false negative rate. The upward (percentage of patients requiring treatment incorrectly identified by the Pima CD4 as above the threshold) and downward (percentage of patients not requiring treatment incorrectly identified by the Pima CD4 as below the threshold) misclassification rates were calculated. The Q-statistic was calculated [35] to quantify and account for the presence of any study heterogeneity due to differences in sample size, study quality, study designs, and/or data collection methods. A bivariate and/or univariate random effects model was applied using METANDI commands in STATA 13.
**(b)** Numerical data analysis	
Methods applied *(where applicable, 95 % confidence intervals (CI) were reported)*	Description
Data description.	The CD4 count paired observations were described by mean (using random effects models), median and standard deviation (SD).
The agreement between the Pima CD4 and reference technology was measured using the Bland-Altman (bias [or mean difference] and SD of the bias) [23],	The Bland-Altman measures the difference between observation pairs (a-b), where method ‘a’ is the Pima CD4. The mean paired difference (the bias or accuracy) and SD of this bias (precision) were determined. A zero mean difference implies good accuracy between reference and Pima CD4 and a small SD of the bias implies good precision (low variability). The accuracy and precision are visually represented on a modified Bland-Altman difference plot with the paired difference on the vertical axis and the absolute CD4 count of the reference on the horizontal axis.
The agreement between the Pima CD4 and reference technology was also measured using the percentage similarity (mean, SD and coefficient of variation [CV]) [24],	The percentage similarity is calculated as the average between the reference and Pima CD4 technology represented as a percentage of the reference technology: [([a + b]/2) /b] × 100, where ‘b’ is the reference method. Observation pairs with the same value will be 100 % similar (accurate) and observation pairs where the Pima CD4 is greater than the reference will be > 100 %, and conversely <100 % if Pima CD4 has a value smaller than the reference. The amount of variability (precision) is represented by the percentage similarity SD and overall agreement by the percentage similarity CV.
The agreement between the Pima CD4 and reference technology was also measured using the percent difference (bias, SD) [25]	The percentage difference is calculated as (a-b)/b (or the average between ‘a’ and ‘b’) × 100 % [25]. Observation pairs with the same value will have no difference and therefore low percent difference, as the percentage difference method is more relative than absolute difference over the range of data.
The strength of the agreement (accuracy and precision) was measured by the concordance correlation (Pc) between the Pima CD4 and reference technologies [17, 36]	The formula applied is pc (concordance correlation) = p (Pearson correlation [measure of precision]) x Cb (bias correction factor [measure of accuracy]) [17, 36]. The value of pc (strength of agreement) is suggested as: <0.9 (poor); 0.90-0.95 (moderate); 0.95 – 0.99 (substantial); >0.99 (almost perfect) [17, 36].
**(c)** Subset analysis	
Description of subset	Methods applied
Sample size in method comparison: Few CD4 method comparison studies’ sample sizes are based on statistical criteria, but rather constrained by costs. This pooled meta-analysis data set afforded the ability to investigate potential impact of sample size on statistical outcomes. An analysis was therefore performed on a subset of data from the comparison between the Pima CD4 and FACSCount of venous derived specimens, as this was the largest subset of paired observations from a single reference and Pima CD4 comparison.	Once the data pairs were entered in MS Excel, random sample numbers (between 1 and 3,486) and irrespective of CD4 category were generated for each CD4 observation pair. This would ensure selection of sample sizes would be independent of the CD4 count and range of CD4 count. The misclassification and agreement analysis was then performed in STATA for sample sizes ranging from 50 to 4,000. The bias, SD of the bias, percentage similarity mean and SD, total misclassification, sensitivity and concordance correlation were all plotted against sample size to determine the impact of sample size on method comparison parameters.
Performance of the Pima CD4 compared to various reference technologies.	The data were sorted based on the reference CD4 method comparator performed in comparison to the Pima CD4, irrespective of study, region or year when the study was performed. The data selection, however, took into account the outcome of the analysis performed in (c) on sample size. Categorical and numerical statistical analyses were applied and results visualized in scatter plots and bar charts.
Performance of the Pima CD4 by different cadre of staff	A subset of 3,751 paired observations was evaluated for total misclassification rates based on different healthcare worker cadres of Pima CD4 operators. This subset was from 11 studies that provided such information with their data. Three cadres were defined: laboratory technician/technologist (includes scientists); laboratory assistant (a lower level of training than technicians) and clinical staff (includes nurses and lay counselors).

## Results

### Data characteristics

**Fig. 1:**
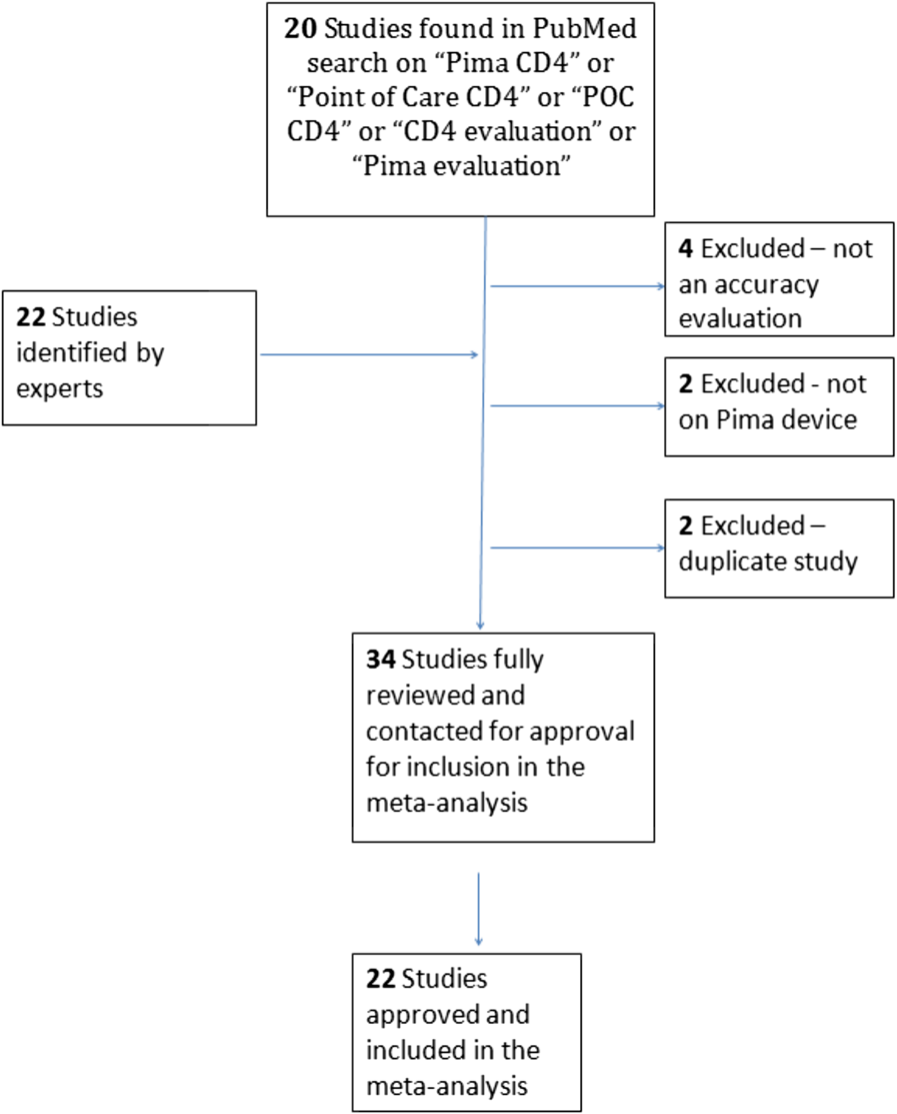
A PRISMA flow diagram of study identification and selection

**Fig. 2:**
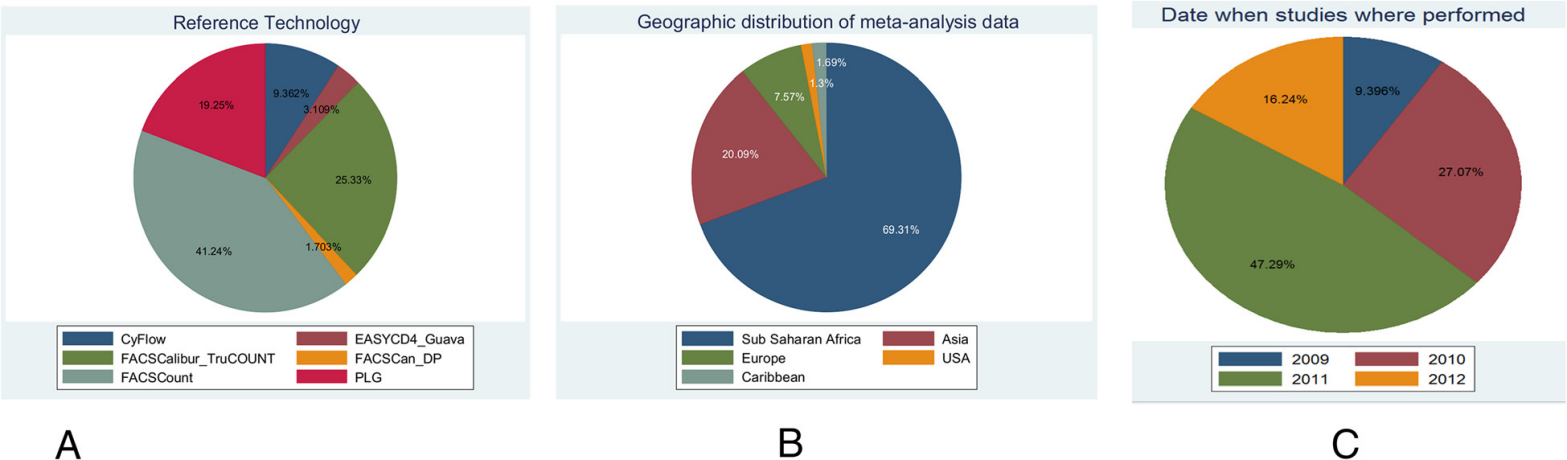
Tabulation of study characteristics and observations summarized in pie charts after sorting by (**a**) comparator reference technologies; (**b**) geographic location of collected observations; (**c**) year in which observations reported

**Fig. 3:**
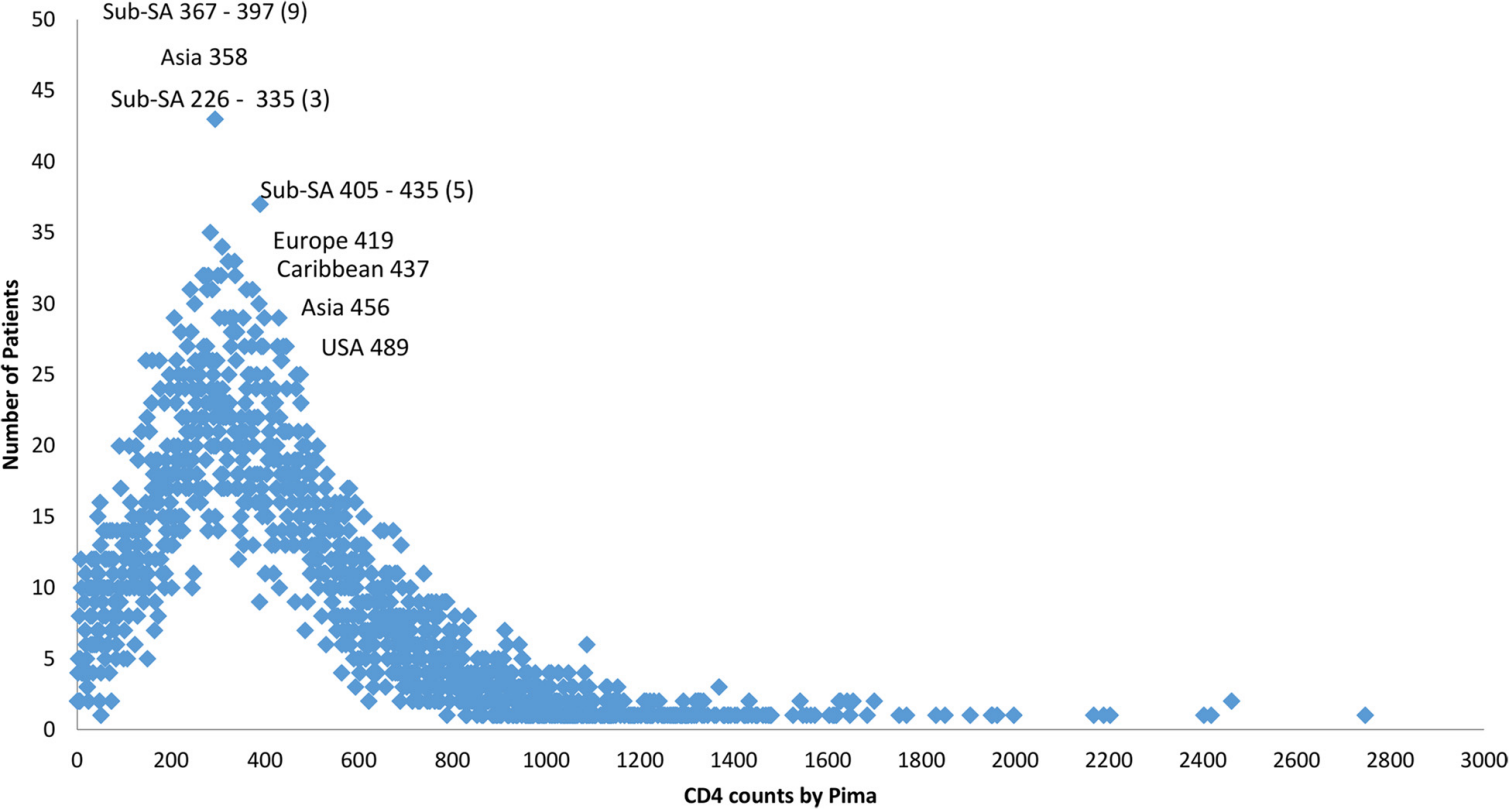
Distribution of CD4 count results generated by the Pima across all the studies. The vertical axis is number of specimens and the horizontal axis is increasing CD4 count. The number of studies from different geographic regions with various median CD4 counts is shown in textboxes on the plot. *Sub-SA* Sub-Saharan Africa

A PRISMA flow diagram (Fig. 1) outlines 34 studies suitable for inclusion in the meta-analysis, and 22 research studies agreeing to participate in this meta-analysis and providing consent for primary data inclusion. These consisted of 10 published studies, 3 studies currently under publication submission and 9 in-country (regulatory) studies. All of these studies investigated the performance of the Pima CD4, either in the laboratory or in health care facilities. Figure 2 illustrates that among the six reference technologies employed across all studies the FACSCount was the predominant reference comparator used in 41 % of the observation pairs. Observations were collected from five global regions between 2009 and 2012 with 62.7 % of the observation pairs from five studies. Overall 69 % of the observations were obtained from studies conducted in sub-Saharan Africa and 47 % of observations were obtained from studies performed in 2011. The distribution plot in Fig. 3 illustrates that the observation pairs have a broad range of CD4 counts from 1 cell/μl to 2,580 cells/μl as reported by the Pima CD4, with a median CD4 count of 363 cells/μl. The median CD4 count for all reference technologies was 383 cells/μl. Of the total number of observations, 65 % were tested by Pima CD4 using venous-derived blood specimens, with a median CD4 count of 373 cells/μl. The median CD4 count of the Pima CD4 using capillary-derived specimens was 342 cells/μl, and studies performed in 2009 had a lower median CD4 count (290 cells/μl) generated from the Pima CD4 than reference technologies (352 cells/μl) or Pima CD4 median results generated in 2010 (327 cells/μl) compared to reference technologies (357 cells/μl). This may indicate changes or improvements in the Pima CD4 technology over time.

### Categorical data analysis

The percentage contribution of observations in the four CD4 categories (<100 cells/μl; 100 – 350 cells/μl; 350–500 cells/μl and >500 cells/μl) as determined by the Pima CD4 and reference technologies found the Pima CD4 had more observations (48.2 %) with CD4 counts <350 cells/μl than reference technologies (44.0 %). In addition, more observations had CD4 counts <350 cells/μl from capillary derived (51 %) than venous derived (46 %) specimens. The proportion test indicated a significant difference (p < 0.001) between the Pima CD4 and reference technologies in the overall numbers of observations in all categories except the 350–500 cells/μl category (p = 0.243). This was similarly found among capillary derived specimens. Venous derived specimens showed no significant difference in the 0–100 cells/μl (p = 0.148) and 350–500 cells/μl (p = 1.06) category assignment by the Pima CD4 compared to reference technologies.

A subset of 584 paired observations from two studies [15, 16], that tested the performance of the Pima CD4 with specimens from HIV treatment-naïve patients, was analyzed to ensure that the results found in this pooled data meta-analysis (n = 11,803) can be applied to this critical population. This would also be useful to determine if changes in clinical thresholds for ART eligibility criteria (350 cells/μl clinical change [17] to 500 cells/μl [2]) using the Pima CD4 would differ from the above analysis. The percentage difference for naïve and meta-analysis observation pairs showed little difference: <100 cells/μl (1 % versus 0.6 %); 100–350 cells/μl (4 % versus 3.6 %); 350–500 cells/μl (−2 % versus 0 %) and >500 cells/μl (−3 % versus −4 %). The Pima CD4, therefore, performed comparably to the reference CD4 technologies overall and in each CD4 category in a subset of HIV-positive treatment-naive patients.

The overall sensitivity of Pima CD4 compared to all reference technologies at all clinical thresholds analyzed was >86 %, and improves at higher CD4 cell counts with sensitivities above 93 % at the two ART initiation thresholds of 350 cells/μl and 500 cells/μl (Table 2). The 95 % confidence intervals for sensitivity at the 350 cells/μl and 500 cells/μl clinical thresholds overlapped between venous and capillary derived specimens, showing this technology has similar performance on venous or capillary derived specimens for identifying patients below these two clinical thresholds. The overall specificity of the Pima CD4 compared to reference technology was >78 % across the three clinical thresholds measured with the lowest specificity observed at the higher CD4 count threshold of 500 cells/μl. The latter comprised 15.5 % of the total number of observations with Pima CD4 count values close to this threshold.

**Table 2:** Categorical meta-analysis summary including random effects modeling

	Overall	Venous	Capillary
	n = 11,803	n = 7,648	n = 4155
Reference Technology			
Mean (absolute range)	428 (402–453)	436 (418–474)	411 (384–437)
Median (IQR)	383 (249–555)	390 (254–565)	371 (241–537)
Pima			
Mean (absolute range)	404 (373–425)	416 (388–444)	382 (351–412)
Median (IQR)	363 (234–524)	373 (242–534)	342 (221–507)
Misclassification			
100 cells/μl			
False positive	1.4 % (0.9 % - 2.0 %)	1.1 % (0.9 % - 1.5 %)	2.1 % (1.3 % - 3.3 %)
False negative	1.0 % (0.7 % - 1.4 %)	0.8 % (0.6 % - 1.0 %)	1.6 % (1.1 % - 2.4 %)
Total misclassification	2.3 % (1.7 % - 3.1 %)	1.8 % (1.5 % - 2.2 %)	3.5 % (2.4 % - 5.0 %)
Upward misclassification	1.5 % (1.0 % - 2.2 %)	1.2 % (0.9 % - 1.6 %)	2.2 % (1.3 % - 3.6 %)
Downward misclassification	14.3 % (11.2 % - 18.1 %)	11.9 % (9.1 % - 15.3 %)	21.0 % (16.1 % - 27.0 %)
350 cells/μl			
False positive	7.5 % (5.9 % - 9.4 %)	6.3 % (4.6 % - 8.6 %)	9.3 % (7.3 % - 11.7 %)
False negative	2.9 % (2.2 % - 3.8 %)	2.3 % (1.7 % - 3.2 %)	3.9 % (2.8 % - 5.3 %)
Total misclassification	11.0 % (9.6 % - 12.5 %)	9.2 % (7.5 % - 11.1 %)	13.8 % (12.1 % - 15.8 %)
Upward misclassification	6.7 % (5.1 % - 8.6 %)	5.7 % (4.1 % - 7.9 %)	8.2 % (5.9 % - 11.2 %)
Downward misclassification	13.7 % (10.9 % - 17.2 %)	10.9 % (8.0 % - 14.6 %)	17.9 % (14.1 % - 22.5 %)
Cadre of staff analysis at 350 cells/ul			
Clinical	n = 1133, 12.0 % (4.7 % - 14.9 %)	n = 510, 11.5 % (7.2 % - 17.8 %)	n = 623, 12 % (9.3 % - 15.3 %)
Lab Assistant	*n* = 558, 12.1 % (9.1 % - 15.9 %)	*n* = 254, 6.6 % (2.2 % - 17.9 %)	n = 304, 15 % (6.3 % - 31.9 %)
Lab Technologist/scientist	n = 2060, 9.2 % (7.1 % - 11.9 %)	n = 1850, 8.3 % (6.5 % - 10.7 %)	n = 210, 13 % (7.3 % - 22.1 %)
500 cells/μl			
False positive	6.7 % (5.6 % - 8.1 %)	6.3 % (5.0 % - 7.8 %)	7.5 % (5.8 % - 9.6 %)
False negative	2.6 % (2.1 % - 3.3 %)	2.0 % (1.5 % - 2.7 %)	3.6 % (2.8 % - 4.6 %)
Total misclassification	9.5 % (8.3 % - 10.8 %)	8.3 % (7.0 % - 9.8 %)	11.3 % (9.6 % - 13.2 %)
Upward misclassification	3.9 % (3.1 % - 4.8 %)	3.1 % (2.3 % - 4.2 %)	5.0 % (3.9 % - 6.5 %)
Downward misclassification	21.8 % (18.0 % - 26.1 %)	18.7 % (14.8 % - 23.4 %)	26.3 % (20.7 % - 32.8 %)
Sensitivity			
100 cells/μl	85.7 % (81.9 % - 88.8 %)	88.1 % (84.7 - 90.9 %)	79.0 % (73.0 % - 83.9 %)
350 cells/μl	93.3 % (91.4 % - 94.9 %)	94.3 % (92.1 - 95.9 %)	91.8 % (88.8 % - 94.1 %)
500 cells/μl	96.1 % (95.2 % - 96.9 %)	96.9 % (95.8 - 97.7 %)	95.0 % (93.5 % - 96.1 %)
Specificity			
100 cells/μl	98.5 % (97.8 % - 99.0 %)	98.8 % (98.4 % - 99.1 %)	97.8 % (96.4 % - 98.7 %)
350 cells/μl	86.3 % (82.8 % - 89.1 %)	89.1 % (85.4 % - 92.0 %)	82.1 % (77.5 % - 85.9 %)
500 cells/μl	78.2 % (73.9 % -82.0 %)	81.3 % (76.6 % -85.2 %)	73.7 % (67.2 % -79.3 %)

Confidence intervals quoted are at 95 %

*IQR* interquartile range

The overall sensitivity of the Pima CD4 at 100 cells/μl compared to reference technologies was less (86 %) than its performance at the ART thresholds (sensitivity >93 %). The Pima CD4 may, therefore, have less ability in identifying all necessary patients requiring reflex CrAg testing. There was also a significant difference in this sensitivity between specimens tested from venous (88 %) compared to capillary (79 %) derived specimens, since the CIs do not overlap. Patients, however, not requiring CrAg reflex testing will be correctly identified by the Pima CD4 since the specificity of the Pima CD4 compared to reference technologies is high (98.5 %), and there was no significant difference between type of specimen (venous or capillary).

The impact of the sensitivity and specificity of the Pima CD4 used at the three clinical thresholds was further investigated through the extent of total numbers of patients who would be misclassified (false positive + false negative rates). The total misclassification rate of Pima CD4 was 2.3 %, 11.0 %, and 9.5 % at the 100 cells/μl, 350 cells/μl and 500 cells/μl thresholds, respectively (Table 2). In addition, the false positivity rates were higher across all clinical thresholds indicating that more patients are found eligible for treatment using the Pima CD4 than reference CD4 technology. This relationship was the same irrespective of specimen type; however, there was greater total misclassification with capillary derived blood specimen testing (3.5 % ≤100 cells/μl; 13.8 % ≤350 cells/μl and 11.3 % ≤500 cells/μl) compared to venous derived specimen testing (1.8 % ≤100 cells/μl; 9.2 % ≤350 cells/μl and 8.3 % ≤500 cells/μl).

This is similarly reflected in the downward misclassification rates, where 14 % of patients would be identified by the Pima CD4 as incorrectly requiring treatment at the ART eligibility threshold of 350 cells/μl and up to 22 % at the ART eligibility threshold 500 cells/μl compared to reference CD4 technology. The upward misclassification of Pima CD4 at the two ART initiation clinical thresholds was less: 7 % (at 350 cells/μl) and 4 % (at 500 cells/μl). Both upward and downward misclassification rates were higher among capillary derived specimens.

A subset of the data (n = 3,751 paired observations) was further analyzed to investigate any differences in the Pima CD4’s performance based on cadre of operator. Seventy percent (*n* = 558 laboratory assistant; n = 2,060 laboratory technician/scientist) of the tests were conducted by laboratory technicians and 30 % (n = 1,133) by clinical staff. Table 2 highlights that the total misclassification rate at 350 cells/μl was below 13 % for laboratory assistants, laboratory technicians and clinical staff. Laboratory assistants performing the Pima CD4 using venous-derived specimens had the lowest total misclassification rate (7 %), yet they also had the highest misclassification rate of 15 % performing the Pima CD4 on capillary derived specimens. Clinical staff had similar misclassification rates (12 %) using either venous or capillary derived specimens. All analyses however showed misclassification rates with overlapping CI’s indicating that technical performance of the Pima CD4 does not alter when used by different cadre of operators.

### Numerical data analysis

The overall mean bias (difference) of the Pima CD4 was −23 cells/μl compared to all reference technologies (Table 3) with overlapping 95 % CI between venous and capillary derived specimen testing. The standard deviation of the overall bias (indicator of precision or variability of the mean difference) was +/− 100 cells/μl for this set of observation pairs with a median CD4 count of 383 cells/μl. The overall mean percentage similarity shows that the Pima CD4 had good accuracy (101 %) compared with the reference technologies but more variability (116 % SD) among capillary than venous (67 % SD) derived specimens. The strength of this agreement (concordance correlation) between the Pima CD4 and reference technologies is also shown to be moderate (Pc = 0.934) for venous derived specimens and poor (Pc = 0.874) for capillary derived specimens, with CI’s that do not overlap.

**Table 3:** Method comparison meta-analysis summary using numerical data

	Overall group	Venous	Capillary
	n = 11,803	n = 7,648	n = 4155
Reference technology			
Mean (absolute range)	428 (402–453)	436 (418–474)	411 (384–437)
Median (IQR)	383 (249–555)	390 (254–565)	371 (241–537)
Pima			
Mean (absolute range)	404 (373–425)	416 (388–444)	382 (351–412)
Median (IQR)	363 (234–524)	373 (242–534)	342 (221–507)
Agreement			
Accuracy and Precision (cells/ul)			
Mean bias (Pima - Reference)	−23	−23	−24
Mean bias (CI)	(−22;-25)	(−21; −25)	(−20; −28)
SD bias	106	93	126
Percentage similarity mean %	101	100	103
Percentage similarity SD %	87	67	116
Percentage similarity CV %	86	67	113
Percent bias (SD) >100 cells/μl	n = 11037, −3.26 % (26.4)	n = 7190, −3.1 % (22.5)	n = 3487, −3.54 % (32.3)
Concordance correlation (*Pc*)	0.914 (0.911, 0.917)	0.934 (0.931, 0.937)	0.874 (0.867, 0.881)
Strength of agreement	moderate	moderate	poor
Overall cell variance			
<100 cells/ul^a^		34	73
100-350 cesll/ul^b^		38	51
350-500 cells/ul^b^		33	57
>500 cells/ul^b^		53	79
Percentage bias across all ranges^c^		10 %	15 %

Calculated from ^a^bias SD; ^b^percentage similarity SD; ^c^the average percentage similarity >200cells/ul

*CV* coefficient of variation, *IQR* interquartile range, *SD* standard deviation

**Fig. 4:**
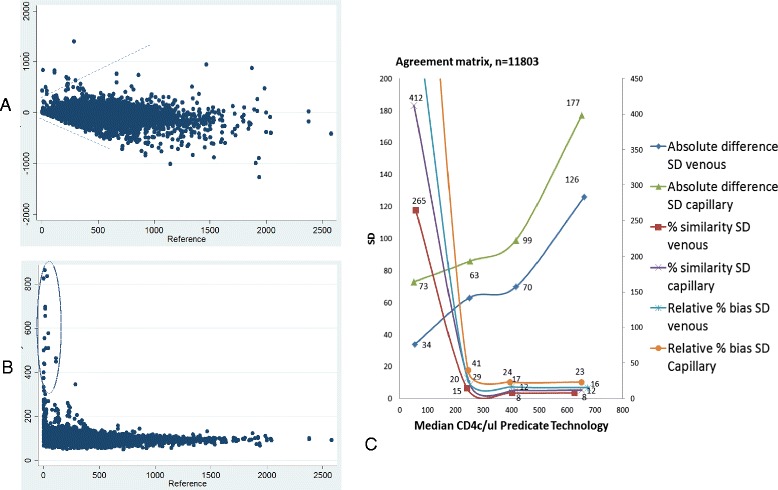
Agreement analysis for 11,803 data paired observations between Pima CD4 and reference CD4 technology testing. Plot (**a**) is a modified Bland-Altman scatter plot with vertical axis as the mean bias (Pima CD4 - reference) and the horizontal axis the absolute CD4 count of the reference technology. The dotted lines illustrate the typical funnel shape of difference not being relative over the range in absolute CD4 counts. Plot (**b**) is the percentage similarity scatter plot with vertical axis the mean percentage similarity values and horizontal axis the absolute CD4 count of the reference. The dotted circle highlights observations pairs that are not clinically relevant outliers in this CD4 count range, but generate high percentage similarity values due to the nature of the method comparison formula. The vertical axis of the percentage similarity plot represents values <1,000. The line plot (**c**) represents the SD of the bias on the vertical axis and the percentage relative bias SD on the secondary vertical axis and the median CD4 count in four CD4 count categories (0–100 cells/μl; 100–350 cells/μl; 350–500 cells/μl; >500 cells/μl) on the horizontal axis. The legend shows the overlay of all three method comparison methods (Bland-Altman, percent similarity and percent bias) for specimens sorted by the specimen extraction method (venous and capillary)

The Bland-Altman, percentage similarity and relative percent bias methods of measuring absolute cell agreement are influenced by certain CD4 count ranges: Bland-Altman by higher CD4 counts and the percentage similarity and relative percent bias by lower CD4 counts. This is visualized in Fig. 4 in the scatter plots by the funnel shape of the Bland-Altman and the outliers in the percentage similarity plots. The line plot in Fig. 4 therefore combines all three agreement measurements by representing their SD (amount of variability) in the four CD4 cell range categories based on reference CD4 technology values. Using the absolute cell difference SD for the <100 cells/μl category and the percent similarity SD and/or the relative percent bias SD for the >100 cells/μl categories shows that the Pima CD4 has good overall agreement with reference CD4 technologies from venous derived specimens, and more variability among capillary derived specimens. The latter is more visible among the absolute difference line plot. Table 3 summarizes this overall agreement (accuracy and precision) from the combined agreement measure methods at the three clinical thresholds. Among venous derived specimens, the cell variance ranges from 34 to 53 cells/μl and among capillary derived specimens it ranges from 51 to 79 cells/μl. Capillary derived specimen testing however has twice as much cell variance (73 cells/μl) than venous derived specimens (34 cells/μl) at the 100 cells/μl clinical threshold, which is also reflected by the significantly reduced sensitivity, 88.1 % (CI 84.7; 90.9 %) vs 79 % (CI 73.0 %; 83.9 %) (Table 2) as previously mentioned. The overall average percent bias of the Pima CD4 compared to reference technologies is 10 % for venous derived specimens and 15 % for capillary derived specimens.

### Performance of the Pima CD4 compared to various reference technologies

**Fig. 5:**
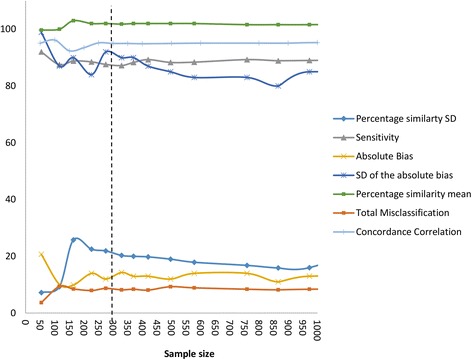
Line plot of method comparison parameters over a range in sample size using observation pairs from the comparisons across studies where FACSCount was the reference CD4 technology compared to the Pima CD4 using venous derived specimen results. The vertical axis has a limit of 100 to accommodate both absolute and percentage method comparison parameters, and the concordance correlation is represented as a percentage. The maximum sample size illustrated is 1,000 for optimal visualization of parameters at the critical range of variability. Misclassification and sensitivity calculations are at the 350 cells/μl threshold. A vertical dotted line illustrates the average/optimal sample size (280) taking into account the variability of all method comparison parameters

As illustrated in the pie chart in Fig. 2, the meta-analysis data comprised observation pairs of the Pima CD4 compared to six reference CD4 technologies contributing from 1.7 % to 41 % of the data. An impact of sample size on method comparison was therefore investigated in a subset analysis using the Pima CD4 versus FACSCount observation pairs. Figure 5 illustrates the changes in misclassification (at the 350 cells/μl threshold), sensitivity (at the 350 cells/μl threshold), measures and strengths of agreement with increasing sample size using the actual clinical data but randomly selecting different sample sizes to include in the analyses. The strength of this agreement (concordance correlation, *Pc*) remained the most constant in value at a minimum sample size of 226. Misclassification, percentage similarity SD and SD of the absolute bias remained constant at a minimum sample size of 329. The percentage similarity mean remained at a constant minimum sample size of 226, and the absolute bias at a minimum sample size of 370. The sensitivity remained constant at the lowest sample size of 164. Beyond these sample sizes there is little change in the method comparison parameters and therefore interpretation of outcomes. Taking these considerations of variability in method comparison parameters into account, the range in optimal sample size is between 164 and 370, with the average of 280. Samples sizes <200 show the least consistency among the method comparison parameters.

**Fig. 6:**
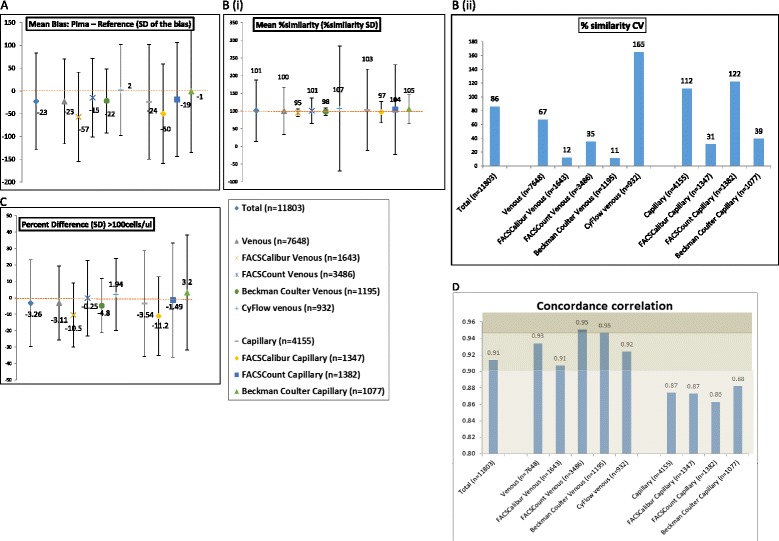
Scatter plots and bar charts of method comparison parameters of the Pima CD4 compared to reference CD4 technologies. Scatter plot **a** = Mean cell bias (Pima – Reference) including standard deviation error bars, with the CD4 count represented on the vertical axis. The dotted line indicates 0 bias. Scatter plot **b**(I) = mean percentage similarity including the % similarity SD, with the vertical axis as % similarity. The dotted line indicates 100 %. Alongside the scatter plot is a bar chart **b**(II) indicating the overall % similarity CV. Scatter plot (**c**) = percent bias (difference) including the percent bias SD for all observations with reference technology values >100 cells/μl, with the vertical axis as % difference. The legend indicates the sample size and the dotted line indicates 0 % difference. Plot **d** is a bar chart representing the concordance correlation between the Pima CD4 and reference technologies. The grey scale shows the strength of agreement (<0.9 = poor; 0.9-0.95 = moderate; 0.95-0.99 = substantial)

Based on this sample size analysis, reference CD4 technologies versus Pima CD4 comparisons (numeric evaluation across all CD4 count ranges) that contributed >370 (maximum) paired observations (from pooled or individual studies) were evaluated to investigate differences in the Pima CD4 compared to the various reference CD4 technologies. The comparisons with the required sample size were: FACSCount (venous n = 3,486, capillary n = 1,382), Beckman Coulter (venous n = 1,195, capillary n = 1,077), FACSCalibur (venous n = 1,643, capillary n = 1,347) and CyFlow (venous n = 932, capillary n = 0). Figure 6 visually represents the method comparison parameters for these evaluations in bias scatter plots (including SD error bars) and bar charts. The mean absolute bias plot (A) indicates the absolute cell bias of the Pima CD4 compared to reference CD4 technologies, ranging from 1 to 57 cells/μl difference. The Pima CD4 has a negative bias (generates lower values) against the FACSCount, FACSCalibur and Beckman Coulter reference CD4 technologies, but generates higher values (positive bias) against the CyFlow. In addition, the Pima CD4 has the lowest variability (SD of the bias) against the Beckman Coulter (SD = 70 cells/μl) reference technology, but this only applies to those specimens tested by venous collection, since capillary tested specimens in contrast generated the greatest variability (SD = 134 cells/μl). The mean percentage similarity plot (B) shows that the Pima CD4 is most similar (least variable) to the FACSCalibur (%SD = 11 % venous derived specimens; 30 % capillary derived specimens), the Beckman Coulter (%SD = 11 % venous derived specimens; 41 % capillary derived specimens) and the FACSCount (%SD = 36 % venous derived specimens) reference CD4 technologies. In addition the Pima CD4 is least similar (most variable) compared to the CyFlow (%SD = 177 % venous derived specimens) followed by the FACSCount (%SD = 127 % capillary derived specimens). The latter is similarly indicated by the high percentage similarity CV values of the Pima CD4 compared to the CyFlow (165 % on venous specimens) and FACSCount (122 % on capillary specimens) as shown in the bar chart (B). These relationships are mirrored in the relative percent bias (difference) plot (C), which represents only the CD4 count range of reference CD4 technology >100 cells/μl, with the least difference (and lowest variability) between the Pima CD4 and the Beckman Coulter (−4.8 %, SD = 16.5 %) and between the Pima CD4 and the FACSCalibur (−10.5 %, SD = 19.5 %) on venous derived specimens. The largest percent difference and variability is between the Pima CD4 and CyFlow (1.94 %, SD = 21.9 %) on venous specimens but even greater percent bias between the Pima CD4 and all capillary derived specimen testing, especially the FACSCount (−1.49 %, SD = 34.8 %) and Beckman Coulter (3.2 %, SD = 35 %). The bar chart in Fig. 6 (d) further illustrates the strength of these agreements between the Pima CD4 and reference CD4 technologies, and shows that the Pima CD4 has substantial agreement (Pc >0.95) with the FACSCount (on venous specimens) and Beckman Coulter (on venous specimens), and moderate agreement (Pc >0.9) with FACSCalibur (venous specimens) and CyFlow (venous specimens) reference technologies. However, the Pima CD4 has poor agreement (Pc <0.9) with all reference technologies when capillary specimens were tested.

## Discussion

This pooled data meta-analysis not only comprises the largest single data set to date published on the performance of a single CD4 enumeration technology, but also comprises observation pairs that are representative of CD4 counts across different geographic regions, observations collected over a fairly short time period (three years) and predominantly (69 %) from high HIV prevalence settings with 55 % from resource-limited settings. There is good representation of six reference comparator technologies that is seldom possible in a single evaluation study. In addition sub-analyses were possible comparing the performance of the Pima CD4 on venous and capillary derived specimens, different cadres of staff and sub-population of HIV ART-naïve patients. The median CD4 (383 cells/μl) from the reference CD4 technology also shows that conclusions drawn from this study can be well applied to the important 350 cells/μl clinical threshold for ART initiation and categorizing this large sample size (11,803) allows for conclusions also to be extrapolated to the 100 cells/μl and 500 cells/μl clinical thresholds. This meta-analysis, therefore, provides a unique opportunity to evaluate the Pima CD4’s technical performance independent of influence from patient age, immunological status, gender, pregnancy, geographic location, HIV status, HIV subtype (by geographic location), instrument, reagent lot, assay version, operator training and sample size that may otherwise influence a smaller study’s analyses.

Overall, the Pima CD4 generates lower CD4 count values than reference technologies with the effect that more patients’ CD4 counts are categorized <350 cells/μl by the Pima CD4, and this is more marked among capillary than venous tested specimens. In absolute cell numbers this equates to an average bias between the Pima CD4 and reference technologies of −23 cells/μl with variability in the bias (SD) across the range in CD4 counts (1-2,800 cells/μl) increasing to SD = 93 cells/μl difference (23 % relative bias and 67 % similarity CV) for venous derived specimens and up to SD = 126 cells/μl difference (32 % relative bias and 113 % similarity CV) for capillary derived specimens. The overall bias across all CD4 ranges may be summarized as 10 % for venous derived and 15 % for capillary derived specimens.

Some variability was noted among the reference technologies (as has been noted by others [18]), with the outlier (higher variability, least similarity) being the Pima CD4 compared to the CyFlow. When the Pima CD4 was compared to the CyFlow reference technology only, this generated a positive bias and the most variability. This may be due to both technologies being based on volumetric testing and using testing volumes <50 μl. The Pima CD4 compared to the Beckman Coulter PanLeucogated reference CD4 technology yielded the least variability, but only among venous tested specimens. This may be due to the Beckman Coulter technology being based on counting total white cells to generate a CD4 count and therefore differences between fresh capillary tested Pima CD4 specimens versus >1 hour old anti-coagulated Pima CD4 and Beckman coulter tested specimens [19]. The Pima CD4 also compared well to the FACSCalibur and FACSCount technologies, but for the FACSCount this was found only for venous derived specimens. The FACSCalibur testing requires a highly skilled operator’s input for manual gating, interpretation and complex software compared to the FACSCount and Pima CD4 which are closed with no operator input to refine the software selection of the CD4 positive cell cluster. The heterogeneity among the reference technologies illustrates the importance of selecting the most appropriate reference technology comparator for such technical evaluations of new technologies. Furthermore, it is critical that the reference technologies meet all quality requirements including participation in external proficiency testing before commencing evaluations.

Improvement in misclassification of the Pima CD4 over time was also noted, and may be due to changes in software, hardware, changes in the type and use of lancets as well as training of operators during implementation. Operator training is key to successful implementation of new technologies. This should be considered for future evaluations of early versions of new platforms that may not be fully optimized, to ensure that promising products are not unduly excluded from consideration for implementation. It is also important for national HIV treatment programs wishing to implement the Pima CD4 (or fast followers) to be aware of allowable differences in bias, resulting in misclassification rates and reduced sensitivity compared to the reference CD4 (or current “in-country”) technology, that may impact treatment costs and weigh the performance and costs with the increased patient access such a technology will allow.

The impact of these differences between the Pima CD4 and reference CD4 technologies was investigated through misclassification and overall sensitivity at three clinical thresholds, summarized in Table 4. Overall, the misclassification by the Pima CD4 compared to reference technology is predominantly due to higher false positive than false negative rates and therefore more patients identified for treatment using the Pima CD4. This results in greater downward misclassification at all thresholds, and is also reflective in the overall specificity of the Pima CD4 of 86 % (CI 82.8 % - 89.1 %) at the 350 cells/μl ART threshold and 78.2 % (CI 73.9 % -82.0 %) at the 500 cells/μl ART threshold with no significant difference between venous and capillary tested specimens. The sensitivity, however, of the Pima CD4 at the 100 cells/μl is 86 % (CI 82 % - 89 %), with a significant difference between venous and capillary derived specimens. Programmatic implications are important to consider when implementing a new testing technology, and these increased false positive and downward misclassification rates mean that more patients will be identified as eligible for treatment. While this will lead to initial increases in overall program costs, treatment is initiated sooner with greater impact on patients’ life years saved [3, 10, 20–22]. It is worth stressing that these implications apply across the different thresholds irrespective of the changes in treatment guideline to the 500 cells/μl threshold, and additional studies can now be undertaken to determine the impact Pima CD4 could have on outcomes and costs.

**Table 4:** Clinical relevance of meta-analysis findings, for venous and capillary derived specimen testing by the Pima CD4

Clinical questions	Venous derived specimen testing	Capillary derived specimen testing
Is Pima suitable for screening for reflex testing of CryAg testing at the 100 cells/μl threshold?	Suitable: 88 % sensitive, Negative bias of 34 cells/μl, 1.8 % total misclassification, Good specificity >97 %	Not suitable: 79 % sensitivity, Negative bias of 73 cells/μl, 3.5 % total misclassification, Good specificity >97 %
Is Pima suitable for identifying patients eligible for ART initiation at 350 cell/μl (WHO 2010 guidelines)?		
	Suitable: >91 % sensitive, Negative bias 38-51cells/μl.	Suitable: >91 % sensitive, Negative bias 38-51cells/μl.
	Expect 9.2 % (6.3 % false positive) total misclassification with specificity of 89 %	Expect 13.8 % (9.3 % false positive) total misclassification with specificity of 82 %, Will increase treatment costs significantly more than venous testing.
Is Pima suitable for identifying patients eligible for ART initiation at 500 cells/μl (WHO 2013 guidelines)?		
	Suitable: >95 % sensitive, Negative bias 53-79 cells/μl	Suitable: >95 % sensitive, Negative bias 53-79 cells/μl
	Expect 8.3 % (6.3 % false positive) total misclassification with 81 % specificity	Expect 11 % (7.5 % false positive)total misclassification with 74 % specificity Will increase treatment costs significantly more than venous testing.

Not only does this analysis highlight some difference in reference technologies, but also in some method comparison parameters. Testing a CD4 blood specimen on the same or on a different platform or test will yield a different CD4 count due to variability in accuracy and precision of both the platforms and tests. Where this variability in the CD4 count becomes important is whether or not the variability becomes clinically relevant and patient management is altered. It is this variability that is investigated in method comparisons and we are beyond the inappropriate use of correlation and linear regression for performing such analyses with CD4 counts [23, 24] but also realize a newer approach using concordance correlation has value in scaling the strength of agreement between two technologies. Appropriate methods reported in the literature for the analysis of continuous values of CD4 counts are the difference [23], the percentage difference [25], the percentage similarity [24] and the ratio [26]. The latter three transform the observation pairs into values that can be compared between studies (even where different samples were tested). Specific parameters from these methods are also more informative than others for interpreting acceptable versus non-acceptable performance limits, for example, the mean bias interpreted with the confidence interval for accuracy and standard deviation of the bias for precision, and both accuracy and precision interpreted in the context of the median CD4 count of the observation set. This pooled data meta-analysis also highlighted the flaws associated with using stand-alone method comparison parameters. The Bland-Altman mean bias is not relative over the range of CD4 counts, especially >100 cells/μl and the percentage similarity and relative percentage mean bias is influenced by outliers (non-clinical) in the <200 cells/μl range. The combination of these method comparison parameters provides a more optimal evaluation across the range of CD4 counts. Analyses such as sensitivity, specificity and misclassification are not typical of CD4 technical evaluations, but in the context of CD4 being used for treatment initiation or screening for reflex testing have proved informative.

The sub-study analyses showed no difference in the Pima CD4 performance in a subset of HIV-positive ART-naïve individuals versus the meta-analysis findings. This was also true for the Pima CD4 testing performed by different cadres of operators. In addition to these findings, the subset analysis of the impact of sample size on method comparison parameters determined an average optimal sample size of 280 paired observations (n = 164 for sensitivity and n = 370 for bias calculation) for analyzing CD4 enumeration technologies. This therefore may be a guide to inform future evaluation studies for minimum sample size requirements for different methods of comparison.

While designing and conducting technical evaluations takes time and significant resources, it is critical to ensure that a technology performs comparably to reference standards. This pooled data meta-analysis implies that immunological population differences do not significantly affect the performance of CD4 diagnostic tests, especially in countries within the same geographic region. Performing a technical evaluation in every country considering a new product would, therefore, lead to significant delays in product approval, implementation in health care facilities, and improving the lives of patients. Thus, a harmonized approach could be attained with one large evaluation across sites and pooled data.

## Conclusion

This meta-analysis focused on a method comparison using CD4 observation pairs, and no qualitative analysis of the Pima CD4 technology itself was investigated. Implementation of POC CD4 technologies will require strengthening of decentralized health care networks, including supply chain, quality assessment and program monitoring. POC CD4 technologies, however, will help achieve the bold goals set out by WHO, UNAIDS, and other global stakeholders of initiating significantly more patients on ART and improving patient access to quality care. In conclusion, this meta-analysis demonstrated that the Pima CD4 platform can generate accurate CD4 counts to be used for ART initiation in both laboratory and non-laboratory settings used by either skilled or non-skilled operators.
